# A Novel Mutation in *CRYBB1* Associated with Congenital Cataract-Microcornea Syndrome: The p.Ser129Arg Mutation Destabilizes the βB1/βA3-crystallin Heteromer But Not the βB1-crystallin Homomer

**DOI:** 10.1002/humu.21436

**Published:** 2011-03

**Authors:** Kai Jie Wang, Sha Wang, Ni-Qian Cao, Yong-Bin Yan, Si Quan Zhu

**Affiliations:** 1 Beijing Tongren Eye Center, Beijing Tongren Hospital, Capital Medical University, Beijing Ophthalmology & Visual Sciences Key LabBeijing, China; 2 State Key Laboratory of Biomembrane and Membrane Biotechnology, School of Life Sciences, Tsinghua UniversityBeijing, China

**Keywords:** cataract-microcornea, βB1-crystallin, *CRYBB1*, βB1/βA3-crystallin, structural stability

## Abstract

Congenital cataract-microcornea syndrome (CCMC) is a clinically and genetically heterogeneous condition characterized by lens opacities and microcornea. It appears as a distinct phenotype of heritable congenital cataract. Here we report a large Chinese family with autosomal dominant congenital cataract and microcornea. Evidence for linkage was detected at marker D22S1167 (LOD score [Z]=4.49, recombination fraction [θ]=0.0), which closely flanks the â-crystallin gene cluster locus. Direct sequencing of the candidate âB1-crystallin gene (*CRYBB1*) revealed a c.387C>A transversion in exon 4, which cosegregated with the disease in the family and resulted in the substitution of serine by arginine at codon 129 (p.Ser129Arg). A comparison of the biophysical properties of the recombinant β-crystallins revealed that the mutation impaired the structures of both βB1-crystallin homomer and βB1/βA3-crystallin heteromer. More importantly, the mutation significantly decreased the thermal stability of βB1/βA3-crystallin but not βB1-crystallin. These findings highlight the importance of protein-protein interactions among β-crystallins in maintaining lens transparency, and provide a novel insight into the molecular mechanism underlying the pathogenesis of human CCMC. © 2011 Wiley-Liss, Inc.

## INTRODUCTION

Congenital cataract is a clinically and genetically heterogeneous group of eye disorders that accounts for one-tenth of the cases of childhood blindness (Gilbert, et al., 1993). Its prevalence is estimated to vary from 0.6 to 6 per 10,000 live births (Reddy, et al., 2004). Congenital cataract tends to be inherited in a Mendelian mode, and can be isolated or occur in association with other ocular or systemic diseases. Congenital cataract-microcornea syndrome (CCMC; MIM# 116150) appears as a distinct phenotype affecting 12%-18% of heritable congenital cataract cases (Hansen, et al., 2007), and characterized by lens opacity and a corneal diameter smaller than 11 mm (Easty, et al., 1999). Genetically, CCMC is a heterogeneous condition. To date, up to eight genes have been reported in autosomal dominant CCMC (Kloeckener-Gruissem, et al., 2008; Abouzeid, et al., 2009; Zhang, et al., 2009; Zhou, et al., 2010), including five crystallin genes (namely αA-crystallin (*CRYAA*), βA4-crystallin(*CRYBA4*), βB1-crystallin (*CRYBB1*), γC-crystallin (*CRYGC*) and γD-crystallin (*CRYGD*)), connexin 50 gene(*GJA8*), basic leucine zipper transcriptional factor (*MAF*), and solute carrier, *SCL16A12* (a carboxylic acid transporter). However, the molecular mechanisms of CCMC caused by these gene mutations are still unclear. Functional analysis of mutant gene product is needed to disclose the underlying pathogenesis of CCMC.

In this study, we identified a novel missense mutation, p.Ser129Arg, in exon 4 of *CRYBB1* (MIM# 600929) in a Chinese family with automomal dominant CCMC. Biophysical studies of the recombinant β-crystallins revealed that this mutation resulted in an alteration of the structures of both βB1-crystallin homomer and βB1/βA3-crystallin heteromer. The structural modification led to a significant decrease in the ability of βB1-crystallin to protect βA3-crystallin in the heteromers against aggregation induced by heat. To our knowledge, this is the first mutation in exon 4 of *CRYBB1* found to be disease-causing for CCMC, and is the first identified mutation that lead to the destabilization of proteins by interfering β-crystallin interactions.

## MATERIALS AND METHODS

### Clinical assessment

This study adhered to the tenets of the Declaration of Helsinki and was approved by the ethics committees for medical research at Capital Medical University. A five-generation family with congenital cataract and microcornea, originating from Hebei province, was recruited at Beijing Tongren Eye Center, Beijing Tongren Hospital (Capital Medical University, Beijing, China). A total of 23 family members (9 affected and 14 unaffected) participated in this study and had a full ocular assessment to document the phenotype, including visual acuity testing, slit-lamp photography, corneal diameter measurement, and axial length measurement using A-scan ultrasonography. Following informed consent, peripheral venous blood was collected for genomic DNA extraction using QIAamp DNA kit (Qiagen, Valencia, CA).

### Linkage analysis

Genotyping and allele sharing exclusion analysis was performed as described by Gu et al previously (Gu, et al., 2008). Haplotypes were generated using the program Cyrillic 2.1. A two-point linkage was calculated with the Linkage package (version 5.2). The cataracts in this family were analyzed as an autosomal dominant trait with full penetrance and a gene frequency of 0.0001. The allele frequencies for each marker were assumed to be equal in both genders. The marker order and distances between the markers were taken from the UCSC database (http://genome.ucsc.edu).

### Mutation and bioinformatics analysis

Nucleotide numbering reflects cDNA numbering with +1 corresponding to the A of the ATG translation initiation codon in the reference sequence, according to journal guidelines (http://www.hgvs.org/mutnomen). The initiation codon is codon 1. Mutation analysis of *CRYBB1* (RefSeq NM_001887.3), *CRYBB2* (RefSeq NM_000496.2), *CRYBB3* (RefSeq NM_004076.3), and *CRYBA4* (RefSeq NM_001886.2) was undertaken by direct sequencing using primers and the protocol described previously (Wang, et al., 2007). The possible functional impact of an amino acid change was predicted by the PolyPhen program (http://genetics.bwh.harvard.edu/pph/).

### Plasmid constructs and site-directed mutagenesis

Human lenses total cDNA was obtained using the standard methods as described previously (Gu, et al., 2008). The coding sequences of human βB1- and βA3-crystallin were isolated from the human lens cDNA library by PCR using the following primers: βB1-crystallin (F: CGGGATCCATGTCTCAGGCTGCAAAGGC, R: CCAAGCTTTCACTTGGGG GGCTCTGTGG), βA3-crystallin (F: AAGGATCCATGGAGACCCAGGCTGA, R: GGAAGCTTCTACTGTTGGATTCGGCGAA). The PCR product was cloned in T-simple vector (Takara Corp.) and sequenced. The insert was then cloned in pET28a for protein expression. The sense and anti-sense primers for the construction of the S129R mutant of βB1-crystallin were ACACATGGTCGAGAAGCTACCGCA and TGCGGTAGCTTCTCGACCATGTGT, respectively.

### Protein expression and purification

Details regarding the overexpression and purification of the recombinant proteins were similar to those described previously (Pang, et al., 2010) with some modifications. In brief, the expression constructs were transformed into *E. coli* strain BL21 (DE3). The six-His Tag sequence of pET28a vector was fused to the N-terminus of the genes to facilitate further purification. The overexpression of the proteins was induced by the addition of 0.1 mM IPTG when the cell cultures reached an OD value of 0.6-0.8. After shaking for another 4 h, the cells were harvested, resuspended in buffer A (20 mM sodium phosphate, 0.5 M NaCl, 1% PMSF) and lysed by sonication in ice bath. The soluble fraction was separated by centrifugation at 8000 × *g* for 30 min at 4°C. The recombinant proteins containing six His tags at the N-terminus were purified by an affinity chromatographic method using the Ni^2+^ chelating column. The matrix-bound protein was eluted with buffer A containing 250 mM imidazole (pH 7.4), and then purified by gel filtration chromatography using a Superdex 200 HR 10/30 column in buffer B (20 mM sodium phosphate, 0.15 M NaCl, 1 mM DTT, 1 mM EDTA). The protein concentration was determined according to the Bradford method using bovine serum albumin as a standard (Bradford, 1976). Since the proteins were prone to be degraded at the N-terminus after storage at 4°C for several days, all protein samples were freshly prepared for further biophysical research.

### Biophysical characterization of the recombinant proteins

The βB1/βA3-crystallin heteromer was prepared by incubating equimolar of βB1-crystallin and βA3-crystallin at 37°C for 2 h, and the formation of heteromer was identified by size-exclusion chromatography (SEC) and SDSPAGE analysis. The effect of the mutation on the structures of both βB1-crystallin homomer and βB1/βA3crystallin heteromer was evaluated by far-UV circular dichroism (CD), intrinsic and extrinsic ANS fluorescence and SEC analysis as described previously (Pang, et al., 2010). In brief, the far-UV CD was measured on a Jasco715 spectrophotometer using 0.1-cm-pathlength cells. The fluorescence emission spectra were recorded on a Hitachi F-2500 spectrofluorimeter using 1-cm-pathlength cuvettes. The intrinsic fluorescence was measured using an excitation wavelength of 280 nm, while the ANS fluorescence was obtained by excitation at 380 nm. All spectroscopic experiments were performed at 25°C. The samples were prepared in buffer B with a protein concentration of 0.2 mg/ml.

As for the SEC analysis, 100 μl samples were injected into an analytical grade 24 ml Superdex 75 HR 10/30 column equilibrated in buffer B on an ÄKTA purification system with a flow rate of 0.5 ml/min at 4°C. Every 0.5 ml fractions were collected and analyzed by SDS-PAGE.

### Protein stability

Protein thermal aggregation was used to reflect the effect of the mutation on the stability of both βB1-crystallin homomer and βB1/βA3-crystallin heteromer. The aggregation was initiated by incubating the protein samples at 60°C. The aggregation of the proteins was monitored by turbidity (absorbance at 400 nm) as a function of time on an Ultraspec 4300 pro UV/Visible spectrophotometer. The final protein concentration was 0.2 mg/ml. The aggregation kinetics was analyzed by considering the protein aggregation process as a first order reaction, and the following equation could be used for the fitting of the data:


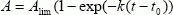
(1)

where *t* is the time of incubation, *A*_lim_ is the *A*_400_ value at the infinite time and *k* is the rate constant of reaction. Data fitting was performed by nonlinear regression analysis using the software Prism, GraphPad Inc.

## RESULTS

### Clinical features of the family

**Figure 1:**
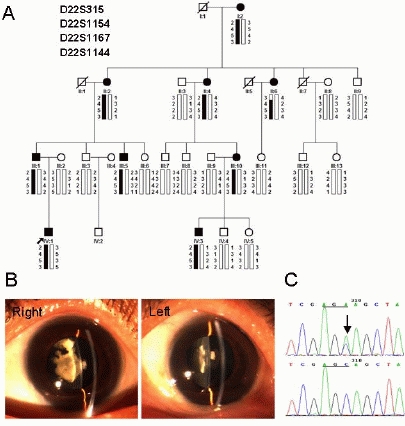
Mutation analysis of *CRYBB1* in a Chinese family with congenital cataract and microcornea. (**A**) Pedigree and haplotype analysis of the family showing the segregation of three microsatellite markers on chromosome 22q11.2-12.1. Squares and circles indicated males and females, respectively. Blackened symbols and bars denoted affected status. The proband was indicated by the arrow. (**B**) Slit lamp photographs of individual II: 2 showing bilateral dense nuclear opacities but asymmetric between eyes. (**C**) DNA sequence chromatograms showed a c.387C>A missense mutation of *CRYBB1* indicated by an arrow. Mutation was numbered according to GenBank NM_001887.3. Nucleotide +1 was A of the ATG initiation codon.

We identified a five-generation Chinese family with clear diagnosis of CCMC (Fig 1A). The affected individuals presented with bilateral dense nuclear opacities affecting the embryonic and fetal nucleus of the lens and average corneal diameter of 10.4 mm. Clinical features of cataract were asymmetric in two eyes of some affected individuals. Individual II: 2 presented with dense round nuclear opacities in left eye, while the other showed irregular nuclear opacities, which extended from the nucleus to peripheral cortex like a tortoise (Fig. 1B). According to the history and medical records, all affected individuals showed cataract within the first year after birth and had the similar poor visual acuity, ranging from 0.05 to 0.2 decimal. They also had nystagmus and amblyopia. Seven of them underwent cataract extraction before the age of 20 years. There was no family history of other ocular or systemic abnormalities.

### Molecular genetic data

Allele-sharing analysis excluded all the known cataract-related loci except β-crystallin cluster on chromosome 22q11.2-12.1. Haplotype analysis showed that the affected individuals in the family shared a common haplotype between D22S1154 and D22S1144 (Fig. 1A). Linkage analysis gave a maximum two-point LOD score of 4.49 for marker D22S1167 at recombination fraction [θ]=0. Mutation analysis of *CRYBB1, CRYBB2, CRYBB3*, and *CRYBA4* in this region showed a single base alteration c.387C>A in the exon 4 of *CRYBB1*, which caused a substitution of serine to arginine at codon 129 (p.Ser129Arg) (Fig. 1C). It was cosegregated with all affected individuals in the family, and was not observed in any of the unaffected family members or 100 normal controls.

The Polyphen score from Polyphen analysis was 1.578, which meant that the p.Ser129Arg *CRYBB1* was predicted to be “probably damaging”.

### Effect of the p.Ser129Arg mutation on the biophysical properties of βB1-crystallin homomer and βB1/βA3-crystallin heteromer

To investigate how the p.Ser129Arg mutation led to CCMC in this large family, the recombinant wild type (WT) and mutant His-tagged βB1-crystallins were produced by overexpression in *E. coli* for further research. Under our experimental conditions, most of the recombinant proteins were found to exist in the soluble fraction after lysis (data not shown), suggesting that both the WT and mutant proteins could fold efficiently in the cytoplasm of the *E. coli* cells. Since βB1-crystallin has been characterized to exist as homomers or heteromers in human lens (Ajaz, et al., 1997; Chan, et al., 2008), the recombinant βA3-crystallin was also purified, and the βB1/βA3-crystallin was prepared by incubating equimolar of βB1-crystallin and βA3-crystallin at 37°C for 2 h. Similar to previous observations (Dolinska, et al., 2009; Srivastava, et al., 2009), the freshly-prepared recombinant proteins mainly existed as a dimer in solutions as identified by static light scattering (data not shown).

**Figure 2:**
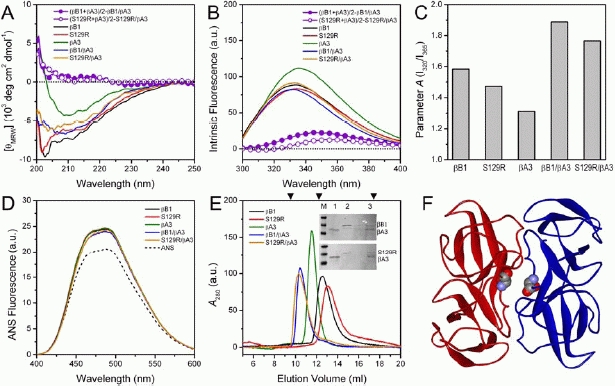
Biophysical characterization of the effect of the p.Ser129Arg mutation on the structures of both βB1-crystallin homomer and βB1/βA3-crystallin heteromer. The samples were prepared by dissolving the proteins in buffer B. The βB1/βA3crystallin was prepared by incubating equimolar of βB1-crystallin and βA3-crystallin at 37°C for 2 h. (A) Far-UV CD. (B) Intrinsic fluorescence. In panels (A) and (B), the difference spectra were produced by subtracting the sum spectra of the homomers by that of equimolar heteromer. (C) Parameter *A*. Parameter *A* was calculated by dividing the fluorescence intensity at 320 nm (*I*_320_) to that at 365 nm (*I*_365_). (D) Extrinsic ANS fluorescence. (E) SEC analysis. The arrow heads along the top axis indicate the elution positions of the standard molecular weight markers of 66 kDa, 29 kDa and 14 kDa, from left to right, respectively. The inset shows the identification of the heteromer by SDS-PAGE of the eluted samples. M, 1, 2 and 3 represents the marker, purified βA3-crystallin, purified βB1-crystallin and the eluted samples of the heteromer collected from the main peak in the SEC profile, respectively. The void volume of Superdex 75 HR 10/30 column is 8 ml. (F) Crystal structure of the truncated βB1-crystallin (PDB 1OKI) (Van Montfort, et al., 2003). The position of Ser129 was highlighted by the space-filling model.

The far-UV CD spectra (Fig. 2A) indicated that the p.Ser129Arg mutation slightly decreased the mean residue ellipticity of βB1-crystallin homomer and the βB1/βA3-crystallin heteromer, suggesting that the mutation led to a minor decrease in the percentages of the regular secondary structure. No significant changes in the secondary structure were observed during the formation of the heteromer for both the WT and mutant βB1-crystallins as revealed by the difference CD spectra (the violet lines in Fig. 2A).

The WT βB1-crystallin contains 8 Trp and 9 Tyr in each subunit, and among them, Trp124 and Trp127 are close to the mutation site. This makes intrinsic fluorescence a powerful tool to monitor the effect of the mutation on its tertiary structure. As presented in Fig. 2B, the WT βB1-crystallin had a maximum emission wavelength (*E*_max_) of ∼334 nm when excited at 280 nm, while a ∼1 nm red-shift of the *E*_max_ was induced by the mutation. When forming heteromer, a blue shift of the *E*_max_ was observed, which was quite consistent with previous observations (Dolinska, et al., 2009; Srivastava, et al., 2009). The alterations in the tertiary structures of βB1- and βA3-crystallins could also be identified by the difference spectra (the violet lines in Fig. 2B), and the mutation affected the structural transitions during the formation of the heteromer. The effects of the mutation could be more clearly characterized by the Parameter *A* analysis, which is a sensitive tool of reflect the position and shape of the intrinsic fluorescence (Su, et al., 2007; Turoverov, et al., 1976). The results in Fig. 2C indicated that the mutation resulted in a change in the shape of the intrinsic fluorescence, suggesting that the mutation slightly modified the tertiary structure of both βB1-crystallin and βB1/βA3-crystallin to a looser one. This deduction is also consistent with the role of Ser129 by structural analysis. Ser129 is located at the domain and subunit interfaces (Fig. 2F) and forms a hydrogen bond with Gly202, but Ser129 is not among the residues that contribute greatly to the stabilization of the domain and subunit interfaces (Van Montfort, et al., 2003). No significant changes in the hydrophobic exposure were observed as reflected by the almost superimposed ANS fluorescence spectra of the 5 proteins (Fig. 2D), which coincides with that Ser129 is on the surface of the dimeric molecule.

The effect of the p.Ser129Arg mutation on β-crystallin quaternary structure was evaluated by SEC analysis. Similar to the previous observations (Dolinska, et al., 2009), the apparent molecular weights of βB1- and βA3-crystallins identified by SEC were lower than the predicted dimer masses, while that of βB1/βA3-crystallin was close to a heterodimer (Fig. 2E). The elution volume of βA3-crystallin was much smaller than that of βB1-crystallin although the calculated molecular weight of βB1-crystallin is larger than that of βA3-crystallin. This observation suggested that the various β-crystallins might have dissimilar shape and/or dimer-monomer equilibrium. The distinct position of the heteromer in the SEC profile suggested that SEC could be used to monitor the formation of the heteromer by βB1- and βA3-crystallin. The mutant S129R eluted at about 0.8 ml later when compared to the WT βB1-crystallin, indicating that the mutant had a relative smaller apparent molecular weight. Static light scattering (SLS) experiments was further conducted to identify the oligomeric state of the proteins. The SLS data indicated that the WT βB1-crystallin mainly existed in a 68.4 kDa form, while the mutant S129R was dominated by a 40.1 kDa form. Thus it seems that the S129R mutation perturbed the dimer interface of βB1-crystallin and led to a shift to the monomeric state in the dimer-monomer equilibrium. However, in the heteromer, the WT and mutant proteins eluted at almost identical volumes. Further analysis of the eluted samples by SDS-PAGE indicated that the mutation did not affect the ability of βB1-crystallin to form heteromer with βA3-crystallin. These observations suggested that the mutation altered the dimer-monomer equilibrium of the βB1-crystallin, but did not significantly influence that of βB1/βA3-crystallin heteromer.

### The p.Ser129Arg mutation destabilizes the structural stability of βB1/βA3-crystallin but not βB1-crystallin

**Figure 3:**
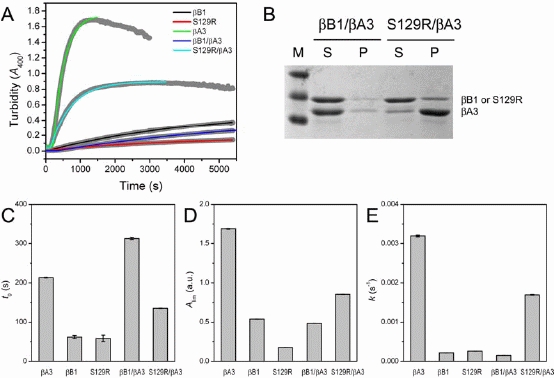
Effect of the p.Ser129Arg mutation on the thermal stability of βB1-crystallin homomer and βB1/βA3crystallin heteromer. (A) The time-course aggregation kinetics monitored by the turbidity at 400 nm at 60°C. The raw data was fitted by Eq. 1, and the kinetic parameters were presented in panels (C-E). The decrease of the turbidity of βA3-crystallin and S129R/βA3-crystallin after long time incubation was caused by the deposition of the large aggregates, and was not included in the curve fitting. (B) SDS-PAGE analysis of the aggregates separated by centrifugation. S and P represent supernatant and precipitate, respectively.

The above biophysical characterization indicated that the p.Ser129Arg mutation affected the structure of βB1-crystallin and βB1/βA3-crystallin differentially at the secondary, tertiary and quaternary structure level. To further investigate whether the changes in the structural features led to an alteration of protein stability, thermal aggregation was used as a model system to reflect the responses of the proteins under stress. The βA3-crystallin homomer was prone to aggregate at high temperatures, while both the WT and mutant βB1-crystallin were rather stable (Fig. 3A). The aggregation of the mutant was less than that of the WT βB1-crystallin as revealed by an about 3-fold decrease of *A*_lim_, but did not affect the values of *t*_0_ and *k* (Fig. 3C-3E). Previous studies have shown that electrostatic interactions play a crucial role in mediating the process of protein aggregation (Chi, et al., 2003; Yan, et al., 2006). Thus the decrease in the amounts of aggregates by the mutation was more like to be caused by the substitution of the polar residue Ser by a charged residue Arg at position 129.

Consistent with previous observations (Srivastava, et al., 2009), the WT βB1-crystallin could efficiently protect βA3-crystallin in the heteromer, and the heteromer was even more stable than both homomers as reflected by a longer *t*_0_ and smaller *k* (Figs. 3C-3E). All the kinetic parameters indicated that the mutant S129R could partially stabilize βA3-crystallin in the heteromer, but the efficiency was much weaker than the WT βB1-crystallin. The SDS-PAGE analysis (Fig. 3B) indicated that most of the proteins were presented in the supernatant for the WT βB1/βA3-crystallin, while the major component in the aggregates of S129R/βA3-crystallin was βA3-crystallin.

## DISCUSSION

In this study, we have identified a novel causative mutation p.Ser129Arg in *CRYBB1* in a large Chinese family with CCMC. βB1-crystallin is a major subunit of the β-crystallins and comprises 9% of the total soluble crystallin in the human lens (Lampi, et al., 1997). The three-dimensional X-ray structure of βB1-crystallin shows it contains two tightly folded domains, N-terminal and C-terminal domains, composed of two Greek key motifs(Van Montfort, et al., 2003). The mutation detected in this family is located in exon 4, which encodes the Greek key II, and replaces the polar uncharged serine by the charged residue arginine at position 129. As suggested by Polyphen analysis, the mutation is predicted to be possibly damaging, which highlights the functional importance of this region of βB1-crystallin. To our knowledge, this is the first report of causative mutations identified in Greek key II of βB1-crystallin associated with CCMC.

Apart from the mutation p.Ser129Arg, totally four mutations in *CRYBB1* have been reported to cause autosomal dominant congenital cataract (ADCC), including p.X253ArgextX27 (Willoughby, et al., 2005), p. Gly220X (Mackay, et al., 2002), p.Gln223X (Yang, et al., 2008) and p. Ser228Pro (Wang, et al., 2007). Interestingly, all sequence changes reported in these ADCC families are located in exon 6, encoding the Greek key IV and the C-terminal extension. These mutations might be predicted to result in an abnormally elongated or truncated COOH-terminus and production of a mutant protein. In addition, clinical phenotypes show some variations among these families but all involved nuclear opacifications to a variable extent. Among them, only one mutation p.X253ArgextX27 has been associated with inherited cataract and microcornea, predicted to elongate the COOH-terminal extension. In ADCC, p.Ser129Arg described in this study is the first mutation of *CRYBB1* identified not in exon 6, which is responsible for bilateral dense nuclear cataract with microcornea and nystagmus in all affected members.

The possible molecular mechanism underlying CCMC caused by the p.Ser129Arg mutation was investigated by comparing the biophysical properties of the WT and mutant recombinant β-crystallins. Unlike the mutations found in the Greek key IV and the C-terminal extension, which resulted in a significant change in the solubility of βB1-crystallin (Mackay, et al., 2002; Srivastava, et al., 2009), we found that the p.Ser129Arg mutation did not affect the folding of recombinant βB1-crystallin in *E. coli*, implying that this novel mutation might undergo dissimilar molecular mechanism with those previously identified ones. Biophysical studies indicated that the mutation modified the structure of both βB1-crystallin and βB1/βA3-crystallin, which is consistent with the fact that Ser129 is close to the domain and subunit interfaces (Fig. 2F) (Van Montfort, et al., 2003). The mutation led to a relatively loose tertiary structure for βB1-crystallin and βB1/βA3-crystallin, but affected their quaternary structure differentially. A pronounced effect was observed on the quaternary structure of the homomer, while no significant change was observed for the heteromer. This suggested that the formation of heteromer might be able to partially rescue the impairment caused by the mutation at the quaternary structure level. It is a pity that the structural basis of this effect was unavailable due to the lack of the high-resolution structure of βB1/βA3-crystallin. A possible explanation is that the homodimer and heterodimer may share different or have alternative subunit interface/sites, as proposed previously (Dolinska, et al., 2009). It seems that the additional sites in the heteromers helped the mutant to maintain the correct quaternary structure under physiological conditions. However, the binding interface was more likely to be weakened by the mutation, and the heteromer might be prone to dissociate under extreme conditions as evidenced by a significant decrease in the stability (Fig. 3).

Many disease-causing mutations of congenital cataracts have been characterized to destabilize the corresponding proteins. Surprisingly, the p.Ser129Arg mutation did not affect the stability of βB1-crystallin, but showed a minor extent of beneficial effects on βB1-crystallin thermal stability. Further research indicated that the mutation significantly decreased the ability of βB1-crystallin to protect βA3-crystallin against aggregation in the heteromer (Fig. 3). βB1-crystallin has long been known to be a crucial protein in forming heteromers with the acidic β-crystallins in lens, and the formation of these heteromers are thought to be important to the maintenance of transparency of the lens (Ajaz, et al., 1997; Chan, et al., 2008). Thus our results strongly suggested that the mutation might result in CMCC via impairing the normal protein interactions and the stability of the heteromers. βB1-crystallins is early expressed during mammal lens development and mainly existed in the nucleus (Aarts, et al., 1989). The mutation might not only affect the stabilities of the β-crystallins, but also result in severe early onset problems in the lens development, which might correlate to the early onset and phenotype of this nuclear cataract shown in Fig. 1. Meanwhile, the expression of β-crystallins are also identified in other eye tissues such as cornea (Clayton, et al., 1986), and the modified protein interactions caused by the mutation might contribute to the development of cornea.

In summary, we have identified a novel heterozygous p.Ser129Arg mutation in *CRYBB1* in a CCMC family of Chinese origin. Biophysical studies indicate that the mutation may lead to CCMC by modifying the structure of βB1-crystallin and disrupting the normal protein-protein interactions, particularly of the interactions of βB1-crystallin with the acidic β-crystallins, which further decreases the structural stability of the β-crystallin heteromers upon stresses. Identification and characterization of this mutation further confirm the importance of protein interactions in maintaining lens transparency, and provide a novel insight into the molecular mechanism underlying the pathogenesis of human CCMC.
